# Suitable Reference Gene Selection for Different Strains and Developmental Stages of the Carmine Spider Mite, *Tetranychus cinnabarinus*, using Quantitative Real-Time PCR

**DOI:** 10.1673/031.010.20801

**Published:** 2010-12-16

**Authors:** W. Sun, Y. Jin, L He, W-C. Lu, M. Li

**Affiliations:** ^1^ Key Laboratory of Entomology and Pest Control Engineering, College of Plant Protection, Southwest University, Chongqing 400716, P. R. China; ^2^ Institute for Control of Agrochemicals, Shandong Province, Jinan 250100, P. R. China

**Keywords:** normalization

## Abstract

Reference genes are used as internal controls in gene expression studies, but their expression levels vary according to tissue types and experimental treatments. Quantitative real-time PCR (qPCR) is the most sensitive technique for transcript quantification provided that gene transcription patterns are normalized to an evaluated reference gene. In this study, the suitability of eight commonly used genes (β?-actin, 5.8SrRNA, α?-TUB, GAPDH, RPL13a, RPS18, TBP, SDHA) were cloned and investigated to find the most stable candidates for normalizing real-time PCR data generated from the four different strains (abamectin-resistant, fenpropathrin-resistant, omethoate-resistant, and susceptible strains) and different developmental stages (eggs, protonymphs, nymphs, and adults) of carmine spider mite, *Tetranychus cinnabarinus* (Boisduval) (Acarina: Tetranychidae). The stability of gene expression was assessed using two different analysis programs, geNorm and NormFinder. Using these analyses, RPS18 and 5.8SrRNA had the most stable expression regardless of the four different strains, whereas RPS18 and α?-TUB were expressed most stably in different developmental stages.

## Introduction

The carmine spider mite, *T. cinnabarinus* (Boisduval) (Acarina: Tetranychidae), is widely distributed all over the world and it is one of the most important and highly polyphagous pests of vegetables and other crops, especially on cotton (LaPlante and Sherman 1976; Hill 1983; Goff 1986; Capinera 2001). For many years the control of *T. cinnabarinus* has traditionally relied on sprays of miticides, and it has been difficult to prevent and control. A major problem in controlling *T. cinnabarinus* is their ability to rapidly develop resistance to miticides after only a few applications (Guo et al. 1998; Herron et al. 1998; Gorman et al. 2001). Given that there will be more studies about mechanisms of resistance to miticides and biochemistry and molecular biology in *T. cinnabarinus,* suitable reference genes are essential for future studies of gene expression using quantitative real-time PCR.

Quantitative real-time PCR (qPCR) has become the method of choice for detection and quantification of mRNA (Bustin, 2000). For accurate gene quantification analysis, normalization of qPCR data is absolutely essential to eliminate template variations between samples due to variations in initial sample amount, mRNA recovery, mRNA integrity, mRNA purity, and reverse transcription efficiency, as well as pipetting errors (Peirson et al. 2003; Wong and Medrano 2005; Nolan et al. 2006). To date, normalization is most frequently achieved by the use of internal controls often referred to as reference genes (Vandesompele et al. 2002), which are used for the normalization of the target gene expression and thereby allow relative expression to be determined. An essential prerequisite for a suitable housekeeping gene is adequate expression in the tissue, but more importantly, the reference gene should show minimal variability and high stability between the normal and experimental conditions. In practice, no single reference gene displays stable expression levels under all experimental conditions, and therefore it is necessary to screen a variety of reference genes under specific experimental conditions for their suitability as internal RNA controls.

In 1999, over 90% of the RNA transcription analyses published in high impact journals used only one reference gene (Suzuki et al. 2000). Vandesompele et al. (2002) described that errors in expression data up to 20-fold can be generated by the use of only a single reference gene. According to Thellin et al. (1999), Vandesompele et al. (2002), Brinkhof et al. (2006), and Peters et al. (2007), at least two or three reference genes should be used for accurate normalization. Furthermore, as several studies have shown that reference genes used for the quantification of mRNA expression can vary with different experimental conditions or tissue types (Thellin et al. 1999; Stüürzenbaum and Kille 2001), each candidate gene should be evaluated before use to make sure it is stably expressed in a particular tissue under the given experimental manipulation. Statistical algorithms such as geNorm (Vandesompele et al. 2002) and Normfinder (Andersen et al. 2004) have been developed to assess the appropriateness of housekeeping genes (Scharlaken et al. 2008).

The aim of this study was to compare expression of candidate reference genes in the four different strains (abamectin-resistant, AbR; fenpropathrin-resistant, FeR; omethoate-resistant, OmR; and sensitive strains, S) and different developmental stages (eggs, protonymphs, nymphs, and adults) of *T. cinnabarinus* to identify those suitable for qPCR studies. Partial sequences of the eight candidate genes (β?-actin, 5.8s rRNA, α?-TUB, GAPDH, RPL13a, RPS18, TBP, SDHA) have been cloned. The stability of the reference genes was assessed by GeNorm (Vandesompele et al. 2002) and Normfinder (Andersen et al. 2004) software packages.

## Materials and Methods

### Insects

Four strains and different instars of *T. cinnabarinus* were used in this study: S, a susceptible strain reared in the laboratory for many years without exposure to insecticides, which was collected from a cropland of cowpea in Beibei, Chongqing, China in 1998; AbR, a abamectin-resistant strain; FeR, a fenpropathrin-resistant strain; and OmR, a omethoate-resistant strain, established by selection with abamectin, fenpropathrin, and omethoate, respectively, for more than 50 generations in the laboratory from the S strain. The age of the developmental stages tested for eggs, protonymphs, nymphs, and female adults were 2d, 0.5d, 1d, and 5d, respectively.

### RNA extraction and cDNA synthesis

Total RNA for the four strains from female adults and different developmental stages from the S strain were extracted with TRNzol Reagent (Tiangen Biotech, www.tiangen.com). For each strain, two hundred individuals were homogenized with at least 1 ml TRNzol Reagent in a glass homogenizer. The process of total RNA extraction and purification were carried out following the manufacturer's instructions including a DNase treatment. Briefly, a maximum of 50 mg of tissue was used per total RNA extraction. These tissues were homogenized using a tissue homogenizer, followed by centrifugation at 12,000 g for 15 min at 4°° C. Total RNA was precipitated from the aqueous phase by adding an equal volume of isopropanol and centrifugation at 12,000 g for 10 min at 4°° C. The total RNA pellet was washed twice with 1 ml of 75% ethanol. Pellets were resuspended in nuclease-free water and the quantity and quality of each sample were analyzed using a SmartSpec™™ 1000 spectrophotometer (Bio-Rad, www.bio-rad.com). All total RNA samples were subjected to DNase digestion in order to remove any residual genomic DNA contamination. Finally the total RNA (A_260_/A_280_=1.8) was dissolved in 40 µµl diethyl pyrocarbonate treated H_2_O and stored at -80°° C for future use. The first strand cDNA was synthesized using 2 µµg of DNase-treated total RNA by RevertAid™™ First Strand cDNA Synthesis Kit (Fermentas life sciences, www.fermentas.com) using the Random Hexamer Primer. The total volume of reverse transcriptional system was 20 µµl (2 µµl dNTP mix (10 mM of each)), 4 µµl 5×× reaction buffer, 1 µµl RiboLock™™ RNase inhibitor (20 U/µµl), and 1 µµl RevertAid™™ M-MuLV Reverse Transcriptase (200 U/µµl)). The procedure was carried out according to the manufacturer's protocol (5 min at 65°° C, 10 min at 30°° C, 1h at 42°° C, and 5 min at 70°° C) and cDNA was stored at -20°° C.

### Reference gene selection, cloning and primer design

Eight reference genes were selected. Primers for β?-actin were used from Xue et al. (2008). The other seven candidate reference genes were drawn from the literature: α?-TUB (Vontas et al. 2000), RPS18 (Donnell et al. 2006), RPLBa, GAPDH, SDHA, and TBP (Vandesompele et al. 2002), except 5.8SrRNA (Table 1). To amplify the partial cDNA fragment of the genes, two degenerate primers were designed by alignment of the amino acid sequences derived from some insects and mites (http://www.ncbi.nlm.nih.gov) *using Primer Premier 5.0* (http://www.premierbiosoft.com/crm/jsp/com/pbi/crm/clientside/ProductList.jsp) *and* *DNAMAN* (http://www.lynnon.com/) (Table 2). PCR reaction was performed with 0.2 µµg of cDNA as a template in PCR buffer containing 3.5 mM MgCl_2_, 0.2 mM dNTPs (deoxynucleotide triphosphates), 0.4 µµM of each primer, and two units of *Taq* polymerase(Takara) in 50 µµl total volume. After an initial 2 min denaturation at 94°° C, 1 min annealing at 50°° C (5.8SrRNA), 51°° C (β?-actin, RPL13a, RPS18), 53°° C (α?-TUB, TBP, SDHA), 49°° C (GAPDH), and 1 min elongation at 72°° C; 35 amplification cycles were performed as follows: 30 s at 94°° C; 40 s at 50°° C (5.8SrRNA), 51°° C (β?-actin, RPL13a, RPS18), 53°° C (α?-TUB, TBP, SDHA), 49°° C (GAPDH); and 1 min at 72°° C; the last extension step was extended to 10 min at 72°° C, followed by cooling to 4°° C, respectively. The products of PCR were analyzed on a 1% low melting point agarose gel. Purified DNA fragments were cloned into the pMD-19T easy vector and transfected into *Escherichia coli* JM109 cells (Takara). Several recombinant clones were identified by PCR amplification and then sequenced with an ABI Model 3100 automated sequencer (Invitrogen Life Technologies www.invitrogen.com). The qPCR primers were designed on the internet (http://www.idtdna.com/SciTools/SciTools.aspx?cat=DesignAnalyze) and the primer parameter set was chosen as ““Real-time PCR””. Primer conditions were optimized by determining the optimal annealing temperature (Ta) and primer concentration (3.125 µµM). Primer and amplicon information are listed in Table 2.

**Table 1.**
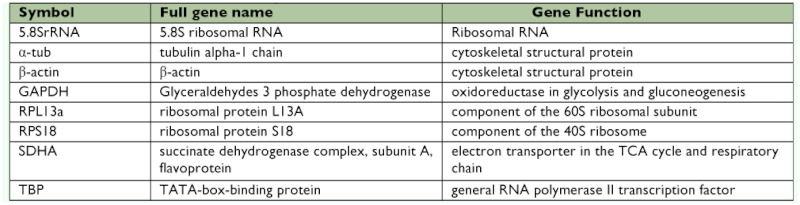
Function of the selected reference genes.

**Table 2.**
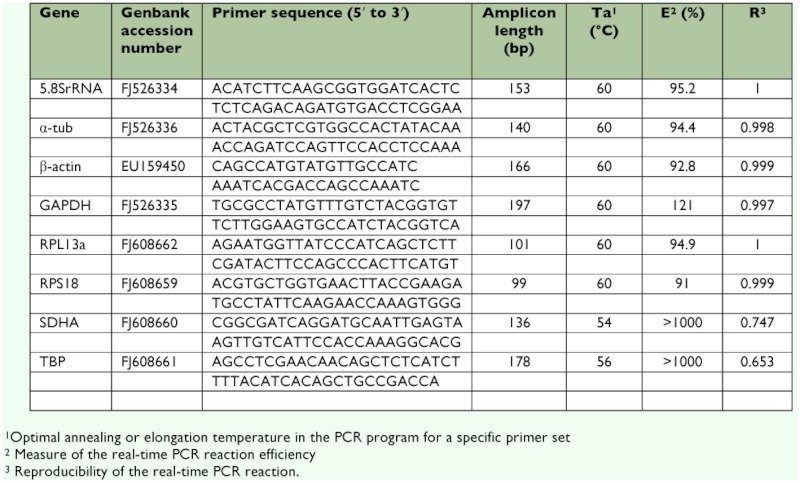
Information of the primers used for real-time PCR.

### Real-time quantitative PCR

The amplification efficiency of each gene was estimated by using the equation, E=10^-1/slope^, where the slope was derived from the plot of amplification critical time (Ct value) versus serially diluted template cDNA concentration. Optimized PCR master mix (20 µµl) contained the following components: 1×× RealMasterMix (including ROX), diluted SYBR Green I (1×× final concentration; Tiangen Biotech), 2 µµl cDNA (equivalent to 10ng total RNA), and 2 µµM sense and antisense primers. The qrtPCR was performed using Mx3000P (Stratagene, www.stratagene.com). Optimized thermal program was 1 cycle of 95°° C for 5min, then 40cycles of 95°° C for 30s, 60°° C for 30s, 68 °°C for 30s, and followed by a final 1 cycle of 60°° C for 30s and 95°° C for 30s. Three negative controls were included in each run. The 4-fold dilution series were used to construct a relative standard curve to determine the PCR efficiency. PCR efficiencies were used to convert cycle threshold values into raw data (relative quantities). Each reaction was run in triplicate, whereby three negative controls were included.

In order to compare the transcription level of the selected genes across different samples and experimental manipulation, the average Ct-value of each treble reaction was converted to raw data for subsequent analysis with the geNorm (Vandesompele et al. 2002) and Normfinder (Andersen et al. 2004) programs.

The geNorm software was used to calculate gene expression stability (M) for each internal control gene tested. For each gene, the program determines the pairwise variation with all other housekeeping genes as the standard deviation of the logarithmically transformed expression ratios. M is the average pairwise variation of a particular gene with all other control genes in a set. A lower M-value denotes a more stable gene expression. The program then performs stepwise exclusion of the gene with the highest M-value (lowest stability) until the two most stable genes are left. Normfinder is another Visual Basic Application, which also assigns a stability value to candidate reference gene. This value ranks the genes using a model based-approach (mixed effect modeling). Instead of analyzing the expression of the whole data set, as is the case with geNorm, this program focuses on the inter- and intra-group expression variations.

## Results

### Transcription profiling of candidate genes

Non-specific amplification can falsely increase gene expression levels and must be avoided, especially when performing real-time PCR using SYBR green intercalating dyes. Nonspecific amplification of the TBP and SDHA genes in *T. cinnabarinus* was confirmed by more than two peaks in real-time melt-curve analysis, so these two genes should be excluded from this study. A standard curve was generated for each gene in *T. cinnabarinus,* using the 3fold serial dilutions of pooled cDNA, generated from susceptive carmine spider mites. The correlation coefficient (R) and PCR efficiency (E) characterizing each standard curve are given in Table 2. PCR efficiencies of the amplification of the eight genes in the mites displayed for most genes very good PCR efficiencies. Also, irrational PCR efficiencies for the genes TBP (>1000%) and SDHA (>1000%) again suggested that these primer pairs should be excluded from further analyses.

### geNorm and NormFinde analyses

The geNorm is a statistical algorithm that was designed to determine the measure of stability (M) for all of the housekeeping genes based on the geometric averaging of multiple candidate genes, as well as the mean pairwise variation of a gene from all other control genes in a given set of samples (Vandesompele et al. 2002).

The genes with the lowest M values will be considered to have the most stable expression of the four different strains (see Table 3) and different developmental stages (see Table 4). As a result, the ranking of gene expression stability value (M) of the six reference genes were presented in Finger 1A and Figure 2A.

**Figure 1.**
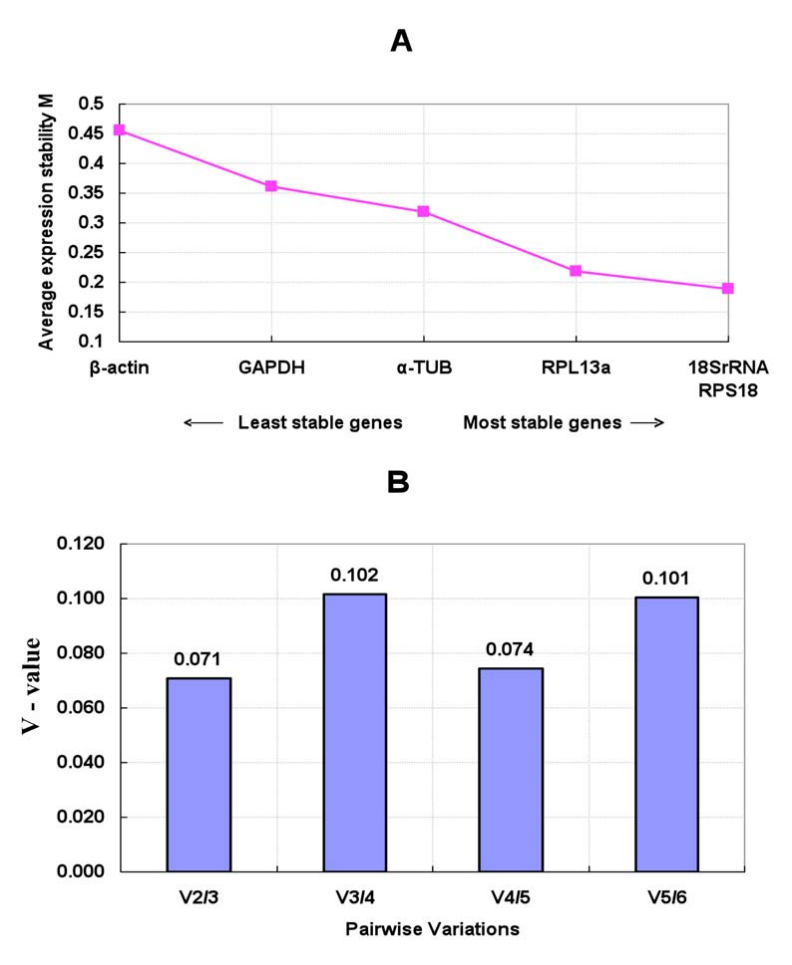
Average expression stability of housekeeping genes tested in different strains of *Tetranychus cinnabarinus* in this study. (A)The average expression stability value, M, was determined using the geNorm program. After removal of the least stable housekeeping gene, the M values of remaining housekeeping genes were recalculated until the last two pairs could not be further compared. Housekeeping genes in order of increasing stability are ranked from left to right, which are indicated by lower M values. (B) Determination of the optimal number of control genes for normalization. Pairwise variation analysis to determine the optimal number of reference genes needed for accurate normalization in V-values less than 0.15 indicates no further genes are needed for calculation of a reliable normalization factor. High quality figures are available online.

**Figure 2.**
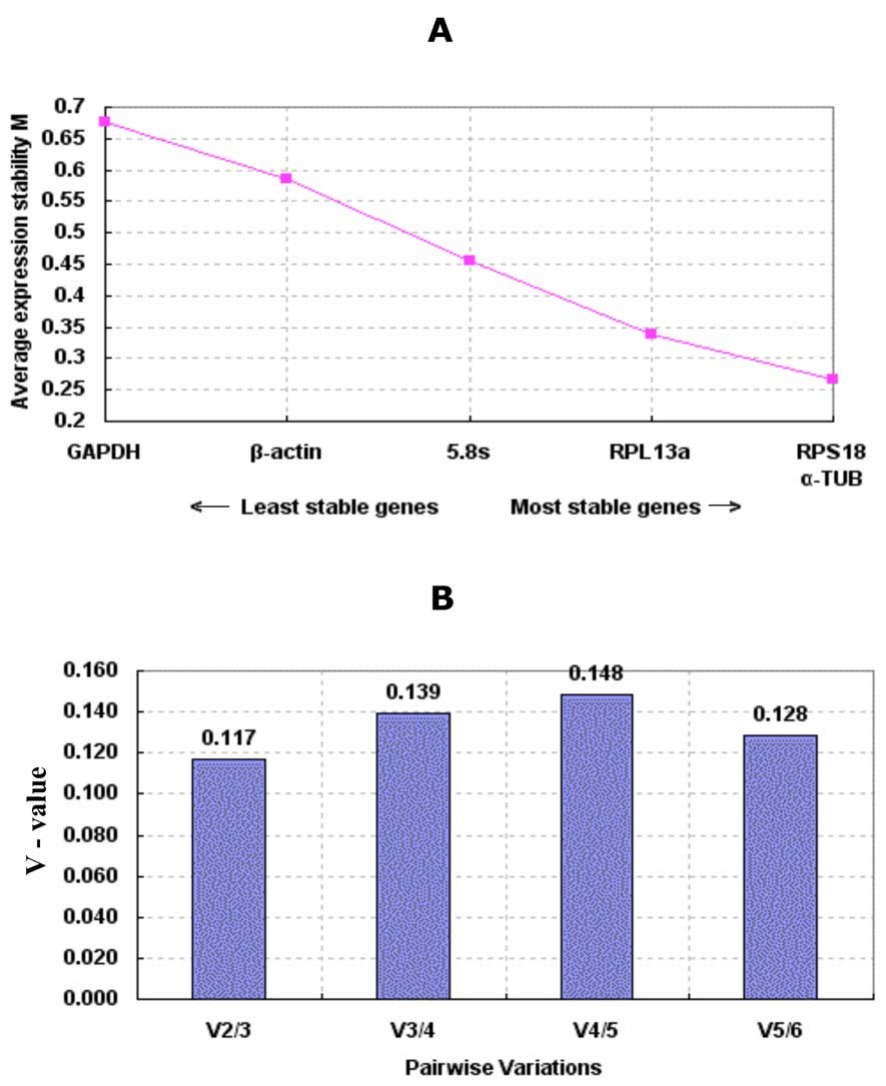
Average expression stability of housekeeping genes of *Tetranychus cinnabarinus* tested in different life stages in this study. (A)The average expression stability value, M, was determined using the geNorm program. After removal of the least stable housekeeping gene, the M values of remaining housekeeping genes were recalculated until the last two pairs could not be further compared. Housekeeping genes in order of increasing stability are ranked from left to right, which are indicated by lower M values. (B) Determination of the optimal number of control genes for normalization. Pairwise variation analysis to determine the optimal number of reference genes needed for accurate normalization in V-values less than 0.15 indicates no further genes are needed for calculation of a reliable normalization factor. High quality figures are available online.

In fact, the normalization with two or more stable reference genes may be required. The geNorm program was subsequently used to calculate the optimal number of reference genes required for the accurate normalization of transcript expression. Pair-wise variations between consecutively determined normalization factors of n and n + 1 genes [Vn/(n + 1)] were calculated (see Figure 1B and Figure 2B). Because the V2/3 values were both below the cutoff value of 0.15 proposed by Vandesompele et al. (2002), the inclusion of a third reference gene would not provide any additional improvement on the statistical significance for each of the housekeeping genes paired groups. From these analyses, two genes with most stable expression, RPS18 and 5.8SrRNA in different strains, and RPS18 and α?-TUB in different life stages of *T. cinnabarinus* were found to be optimal.

To further validate our findings regarding the most optimal reference gene to normalize transcript expression data, the data set was assessed with NormFinder, which was designed to calculate the stability by using the combined estimate of inter- and intra-group expression variations of the analyzed genes. The calculated stability values of the 6 candidate genes were in Table 5 and Table 6. Based on these values, the NormFinder program validated the findings with the geNorm algorithm, in which the most stable single gene was RPS18, and the best combination of the reference genes was RPS18 and 5.8SrRNA in different strains, RPS18 and α?-TUB in different life stages of *T. cinnabarinus.*

## Discussion

The ability to perform accurate normalization is necessary to obtain accurate and reliable results in gene expression studies. Traditionally, only one reference gene is used for normalization purposes in qPCR studies with the most popular being GAPDH, β?-actin, or 18S rRNA (Patel et al. 2002). However, numerous studies have reported that housekeeping gene expression can vary considerably.

Accurate, reproducible, and biologically relevant quantification of transcripts analyzed in quantitative real-time PCR requires data normalization. Otherwise transcript quantities are not comparable, neither between different tissues or developmental stages of the organism itself, or with different, but more or less closely related organisms. The most common and currently preferred method for transcript normalization is the use of internal, evaluated reference genes (Nolan et al. 2006), which are often represented by housekeeping genes. Housekeeping genes to be used as reference genes should meet the three criteria of ubiquitous expression, low variance and a reasonable prospect of not being regulated themselves in the experimental condition under investigation. Practical criteria for the choice of a housekeeping gene are abundance of the transcript, or the fragment size and melting curve of a respective PCR-product (Frericks et al. 2008). Reference gene selection studies dealing with real time PCR normalization strategy usually examined 3–18 genes (Andersen et al. 2004). For these reasons, a panel of housekeeping genes with different transcript abundances and product sizes to choose from would be a useful tool. In this study, 8 genes were chosen and examined, but 2 genes (TBP and SDHA) were excluded before evaluating the stability of expression due to their non-specific amplification. This phenomena also occurred in reference genes choosing for honeybee (Scharlaken et al. 2008), which may indicate the TBP and SDHA were not suitable as reference genes for some species.

**Table 3.**
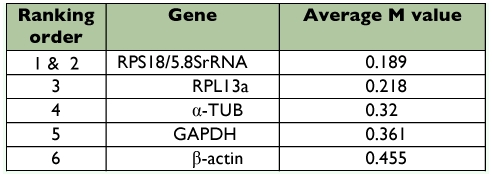
Housekeeping genes for normalization ranked according to their expression stability in different strains (calculated as the average M Value after stepwise exclusion of the worst scoring gene) by geNorm.

**Table 4.**
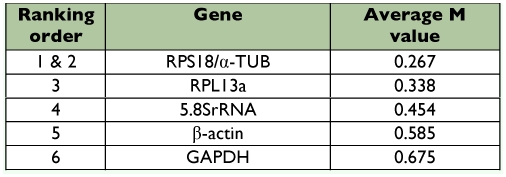
Housekeeping genes for normalization ranked according to their expression stability in different life stages (calculated as the average M Value after stepwise exclusion of the worst scoring gene) by geNorm.

**Table 5.**
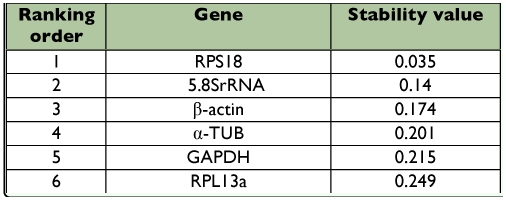
Housekeeping genes for normalization listed according to their expression stability in different strains calculated by NormFinder

**Table 6.**
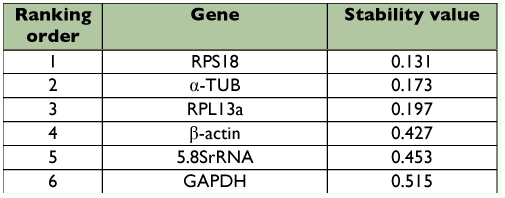
Housekeeping genes for normalization listed according to their expression stability in different life stages calculated by NormFinder.

Raw Ct values were used to evaluate the stability of the housekeeping genes in the experiments using the GeNorm and Normfinder programs. Relative quantification of target genes was performed according to the delta—— delta——Ct method using the indicated the housekeeping genes to normalize results (Balogh et al. 2008).

An additional function of geNorm is to calculate a normalization factor (NFn, n = the number of genes included) for the most stable candidates, then for other genes by stepwise inclusion of the next most stable gene. Afterwards, pairwise variations (Vn/n+1) between two subsequent normalization factors are calculated, indicating the effect of including one additional gene for normalization. If V is large, then inclusion of the subsequent gene for normalization has a significant effect. The recommended cut-off value of 0.15 for V to be significant was used in this study (Vandesompele et al. 2002). When analyzing housekeeping genes for our samples, the inclusion of the two most stably expressed housekeeping genes as reference genes is optimal. Inclusion of additional housekeeping genes for internal control genes beyond the two most stably expressed genes would not have a significant effect on the results.

Both programs identified the same two reference genes as most stable expressed, geNorm provides information about the optimal number of genes in a given experiment, whereas Normfinder gives additional information about the inter- and intra-group expression variations. From the two programs, RPS18 and 5.8SrRNA can be considered as the best reference genes in different strains, RPS18 and α? -TUB as in different developmental stages. It is important to state that these reference genes are not necessarily suitable for use with other tissue types. Reference genes should be detected prior to qPCR studies and the most stable for the specific tissue or condition chosen, as presently demonstrated. In addition, it is important to state that we do not advocate the use of one reference gene, although RPS18 expressed most stably in both different strains and different development stages of *T. cinnabarinus.* It has previously been demonstrated that the use of two to four reference genes improves accuracy of quantification (Peters et al. 2007; Brinkhof et al. 2006; Vandesompele et al. 2002; Suzuki et al. 2000; Thellin et al. 1999).

Although there is a strong possibility that other more suitable reference genes other than the ones analyzed in this study, it has been confirmed that 5.8SrRNA and RPS18 and α?TUB genes gave reliable and stable gene expression compared to other more commonly used housekeeping genes, including β?-actin and GAPDH.

Xue et al. (2008) cloned a fragment of β?-actin gene from *T. cinnabarinus* and tried to establish it as the reference in *T. cinnabarinus,* however, the stability of expression of β?-actin was not examined, although specific amplification was obtained. This paper is the first to describe the evaluation of multiple reference genes for normalizing real-time PCR data generated from in *T. cinnabarinus.* It is also the first, to our knowledge, to conduct such evaluations for Acarine. Although the stability of gene expression for various candidate housekeeping genes can vary greatly during the different strains and different life stages, the statistical analyses based on geNorm and NormFinder software definitively showed that normalizing target gene expression with the combined mean expression values of RPS18 and 5.8SrRNA genes was the most appropriate and accurate method when assessing expression profiling during the different strains in carmine spider mite, RPS18 and α?-TUB are the same as in different developmental stages, which also indicated that using the three references (RPS18, 5.8s, and a-TUB) in carmine spider mite could be as a best-in-all-cases reference in future study.

Although none of the evaluated reference genes was suited for all tissue comparisons, or all experimental conditions, two or more were found to be acceptable in each situation examined excepting a comparison across multiple tissue types. The results of this study emphasize the absolute requirement for validation of real-time PCR data normalization procedures. Basic knowledge of stability and expression of select reference genes across a variety of *T. cinnabarinus* are provided thus serving as a guideline for reference gene selection in other *T. cinnabarinus.* There are many strategies for real-time PCR data normalization not discussed here, but regardless of the strategy used, the selection of reference genes must be properly validated for particular tissue or cell types and particular experimental models for proper interpretation and repeatability. In conclusion, the results obtained from this study will benefit future study of comparing the gene expression in *T. cinnabarinus,* such as the different expression patterns of detoxification enzyme genes between the acaricide susceptible and resistant *T. cinnabarinus.*

## Abbreviations

**AbR**,: abamectin resistant;
**FeR**,: fenpropathrin resistant;
**OmR**,: omethoate resistant;
**qPCR**,: Quantitative real-time PCR
